# Revisiting the Extensor Hallucis Longus Tendon: Anatomical Classification and Orthopedic Implications

**DOI:** 10.3390/jcm14196925

**Published:** 2025-09-30

**Authors:** Łukasz Olewnik, Ingrid C. Landfald, Paloma Aragonés

**Affiliations:** 1 Department of Clinical Anatomy, Mazovian Academy in Płock, 09-402 Płock, Poland; ingridceciliee@gmail.com; 2 VARIANTIS Research Laboratory, Department of Clinical Anatomy, Mazovian Academy in Płock, 09-402 Płock, Poland; paloarag@ucm.es; 3 Department of Orthopedic Surgery, Medical Faculty, Universidad Complutense de Madrid, 28040 Madrid, Spain

**Keywords:** extensor hallucis longus, anatomical variation, tendon classification, foot and ankle surgery, imaging, ultrasound, MRI

## Abstract

**Background**: Anatomical variations of the extensor hallucis longus (EHL) tendon hold significant implications for foot and ankle surgery, yet they remain underrepresented in orthopedic literature. Accurate recognition of these variants is crucial for minimizing iatrogenic injuries and improving surgical outcomes. **Aim**: This narrative review aims to summarize current anatomical knowledge on EHL tendon morphology, with a particular focus on the classification system proposed by Olewnik et al. Emphasis is placed on its diagnostic, radiological, and surgical relevance. **Methods**: A comprehensive literature review was conducted, integrating findings from cadaveric dissections, imaging studies, and clinical observations. The Olewnik classification—based on the number and insertion of EHL tendon slips—serves as the organizing framework for the anatomical and surgical discussion. **Findings**: The Olewnik classification delineates three primary types: Type I (single slip), Type II (two slips, subdivided into IIa–IIc), and Type III (three slips). Each type is discussed in terms of anatomical features, diagnostic challenges on MRI and ultrasound, and implications for surgical exposure, tendon transfer, and graft harvesting. Comparative analysis with prior typologies underscores the enhanced clinical utility of the Olewnik system. **Conclusions**: The reviewed classification offers a reproducible, imaging-compatible, and surgically applicable framework for understanding EHL tendon variability. Incorporating this system into preoperative planning may enhance procedural safety and precision. Further clinical validation and broader integration into surgical education are warranted.

## 1. Introduction

**Figure 1:**
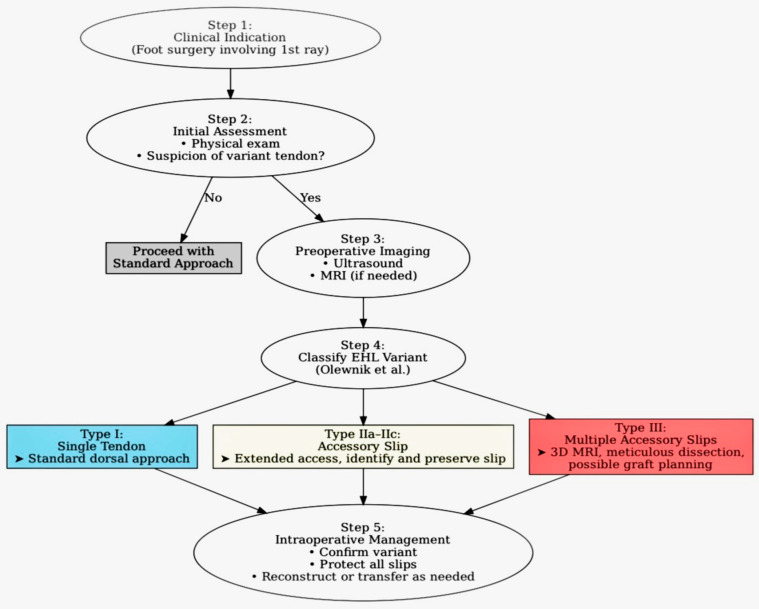
Diagnostic–surgical workflow for extensor hallucis longus (EHL) tendon variants. Step 1: Clinical history and examination. Step 2: Clinical clues that raise suspicion for a variant: palpable or visible additional dorsal cord over the first ray; dorsal pain or snapping with active hallux dorsiflexion; isolated hallux dorsiflexion weakness with preserved EDL function; unexplained hallux extensus (“cock-up deformity”) after trauma or surgery; discordance between clinical findings and a single-slip appearance on initial imaging. Step 3: Targeted imaging. Dynamic high-frequency ultrasound tracking each slip to its insertion; MRI with thin-slice axial and sagittal PD/T2 fat-saturated sequences and, when available, a thin-slice 3D isotropic sequence covering the first ray. Step 4: Classification and reporting according to the Olewnik system (Types I–III; IIa–IIc), including insertion site(s) and relationship to EHB/EDL. Step 5: Surgical planning. Type I: standard dorsal approach. Type IIa–IIc: extended exposure, identify and preserve accessory slip. Type III: consider 3D isotropic, MRI, meticulous dissection, potential graft planning. (Olewnik et al. [1]).

This review presents a comprehensive anatomical classification of extensor hallucis longus (EHL) tendon variants as proposed by Olewnik et al. [1] and explores their clinical relevance for orthopedic surgery, with emphasis on morphological subtypes and implications for procedures on the dorsum of the foot and first ray. The EHL is essential for hallux dorsiflexion and toe clearance during gait, contributing to stabilization during push-off; distal-course and insertional variants can alter surgical exposure and increase the risk of iatrogenic injury, underscoring the need for a standardized framework to guide preoperative planning and intraoperative decisions [2,3]. The literature has progressed from isolated cadaveric reports and case-based imaging observations to structured, large-scale classifications [1,2,4], while US/MRI studies have highlighted diagnostic pitfalls associated with bifid or accessory tendons [5,6]. A unified, surgically actionable classification has only recently been proposed and remains underrecognized in orthopedic literature. Beyond surgical exposure and imaging interpretation, foot-posture research in flexible flatfoot underscores how targeted neuromuscular training can influence first-ray mechanics and balance—context that reinforces the clinical salience of recognizing EHL morphology and its variants [7]. The diagnostic–surgical workflow is summarized in Figure 1.

## 2. Anatomy and Function of the Extensor Hallucis Longus Muscle

### 2.1. Biomechanical Function of the EHL

The EHL originates from the middle third of the fibular shaft and the adjacent interosseous membrane and courses anteriorly to insert on the dorsal aspect of the distal phalanx of the hallux [1,4]. Its primary role is dorsiflexion of the hallux, which is essential during the swing phase of gait for toe clearance, and contributes to stabilization during toe-off. The EHL also assists in dorsiflexion of the foot and contributes to the maintenance of medial longitudinal arch integrity, playing a secondary role in forefoot and ankle biomechanics [8,9].

Beyond simple hallux extension, EHL activity dynamically modulates forefoot positioning and pressure distribution across the first ray. As such, it is intricately involved in maintaining balance during stance and transition between phases of gait [10]. The EHL also contributes to proprioceptive feedback through its muscular-tendinous receptors, enhancing joint stability during ambulation [11].

### 2.2. Clinical Relevance

Due to its anatomical course and biomechanical role, EHL dysfunction can lead to diverse pathologies, including hallux extensus (“cock-up deformity” of the great toe), claw toe, or traumatic rupturę [6,12]. Surgical interventions on the dorsal foot carry a risk of iatrogenic injury, particularly in patients with neuropathy or diabetes, where altered toe clearance may cause secondary complications [5]. Given its use in tendon transfer and grafting, preoperative identification of anatomical variants is essential to ensure optimal outcomes and minimize operative risk [13,14].

## 3. Olewnik et al. [1] Classification

### 3.1. Type I—Classical Configuration

In Type I, the EHL tendon follows a single, uninterrupted course and terminates as an extensor hood inserting onto the dorsal base of the distal phalanx of the hallux [1]. This morphology was observed in 57.7% of the specimens examined in a cadaveric series comprising 102 lower limbs.

Clinically, this configuration is considered the reference type. It aligns with traditional anatomical descriptions and presents minimal risk during operative exposure. Its predictable course allows for straightforward surgical approaches, whether in tendon repair, transfer, or soft-tissue balancing procedures. Moreover, Type I is rarely associated with misdiagnosis in imaging or intraoperative confusion, making it ideal for procedures requiring graft harvest or precise tendon localization [1,4].

### 3.2. Type II—Two Tendinous Slips (Subtypes IIa–IIc)

In Type II variants, the EHL tendon bifurcates distally into two separate slips: a dominant tendon inserting onto the distal phalanx (as in Type I), and an auxiliary tendon with variable insertion. These were observed in a combined 40.4% of cases, subdivided as follows: Type IIa in 29.9%, Type IIb in 4.8%, and Type IIc in 5.7% of limbs [1].

#### 3.2.1. Type IIa

The accessory slip inserts on the dorsal aspect of the proximal phalanx of the hallux, typically medial to the insertion of the extensor hallucis brevis (EHB). This variant may be mistaken intraoperatively for an anomalous EHB tendon, particularly in cases of medial deviation or surgical correction of hallux valgus. Identification on imaging is generally feasible using high-resolution ultrasound or MRI, but may require careful dynamic evaluation [1].

#### 3.2.2. Type IIb

This subtype mirrors the insertion site of IIa but features a bifid configuration of the accessory tendon, often presenting as two discrete slips inserting closely on the proximal phalanx just distal to the EHB. Type IIb carries a heightened risk of misinterpretation as a partial tendon tear or soft-tissue mass on imaging [5]. In surgical settings, the bifid morphology may complicate tendon exposure and dissection, particularly during transfer procedures or correction of hallux deformities.

#### 3.2.3. Type IIc

Here, the accessory tendon deviates further distally and inserts on the dorsal base of the first metatarsal. This variant is rarely visualized intraoperatively unless specifically sought. It poses a challenge in tendon harvesting or reconstruction, as surgeons may unintentionally transect the accessory slip under the assumption of encountering a slip from EHB or extensor digitorum longus. MRI is essential in preoperative recognition and classification of this subtype [15].

Across all subtypes of Type II, the presence of an accessory tendon increases the complexity of both diagnosis and surgical intervention. A common pitfall involves misidentification of the accessory slip as the EHB, especially when insertions lie medially or proximally. Surgeons should anticipate such anatomical variations and integrate high-resolution imaging into preoperative planning to avoid misinterpretation and minimize iatrogenic injury [1,3,6].

### 3.3. Type III—Three Tendinous Slips

Type III is the rarest morphological variant of the EHL tendon, observed in only 1.9% of dissected limbs [1]. It is characterized by the presence of three distinct tendinous slips, each with separate insertions and functional implications. The main slip inserts onto the dorsal base of the distal phalanx of the hallux, maintaining the classical pathway of EHL. A medial auxiliary slip also terminates on the distal phalanx but attaches more proximally than the main tendon, potentially reinforcing dorsiflexion or altering force transmission. The lateral auxiliary slip, typically the more robust of the two accessory tendons, merges with the EHB and inserts on the dorsal aspect of the proximal phalanx. High-resolution 3T MRI or 3D ultrasound is necessary to delineate the complex anatomy preoperatively [6]. Surgically, Type III poses increased risks during tendon transfer, repair, or exposure. Inadvertent transection of an auxiliary slip may result in loss of hallux control or postoperative imbalance. Moreover, the fusion of the lateral slip with EHB can obscure surgical planes and complicate dissection during correction of deformities such as hallux varus or extensus [12]. Due to its low prevalence and anatomical complexity, Type III underscores the importance of detailed imaging and cautious intraoperative assessment in any procedure involving the dorsum of the foot.

**Figure 2:**
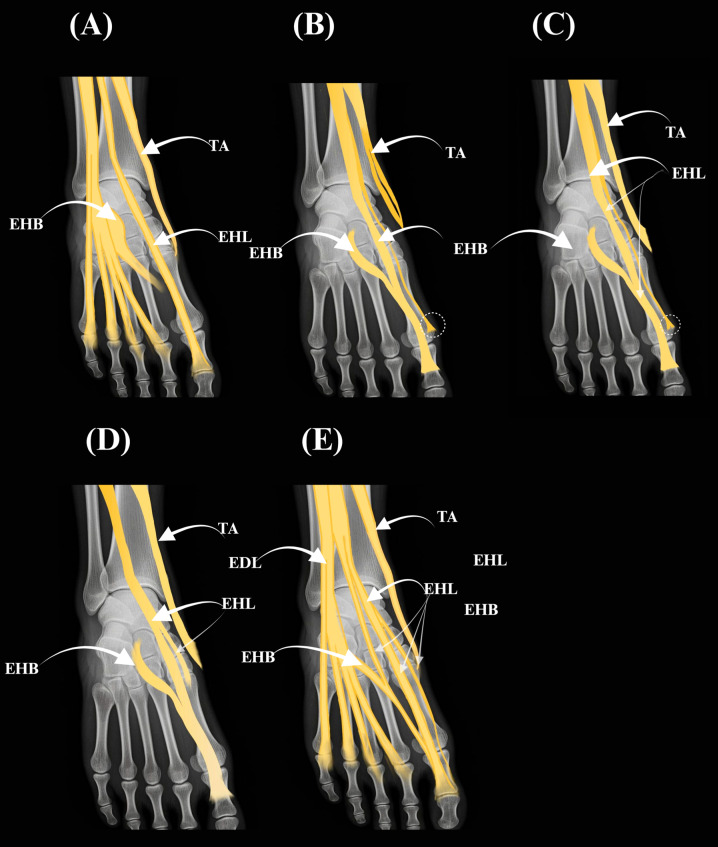
Atlas of extensor hallucis longus (EHL) tendon variants according to (Olewnik et al. [1]) Types I–III with subtypes IIa–IIc are illustrated as simplified overlays on a dorsum-of-foot template. (**A**) Type I (single slip to the distal phalanx). (**B**) Type IIa (two slips; accessory slip inserting into the proximal phalanx; dashed circle). (**C**) Type IIc (two slips; accessory slip inserting into the base of the first metatarsal; dashed circle). (**D**) Type IIb (two slips; bifid accessory at the proximal phalanx). (**E**) Type III (three slips). Arrows indicate insertions and course. Abbreviations: EHB—extensor hallucis brevis; EDL—extensor digitorum longus; TA—tibialis anterior.

A schematic atlas of the Olewnik et al. [1] variants is provided in Figure 2 (panels A–E).

## 4. Imaging Considerations in Diagnosing EHL Variants

An accurate understanding of EHL tendon anatomy is crucial in both diagnostic and preoperative contexts. Given the variability of EHL morphology, imaging plays a central role in the identification and differentiation of anatomical subtypes. While Type I tendons are typically straightforward to visualize, Types II and III require advanced imaging modalities and careful interpretation to avoid misdiagnosis and intraoperative surprises.

### 4.1. Type I

#### 4.1.1. Ultrasound and MRI Features

In Type I variants, the EHL tendon follows a single, predictable trajectory and inserts onto the dorsal base of the distal phalanx. This configuration is readily identifiable on both US and MRI. Standard longitudinal and axial views reliably demonstrate the tendon as a homogeneous, fibrillar structure coursing between the tibialis anterior and extensor digitorum longus, superficial to the first metatarsal and medial cuneiform [6].

On MRI, the EHL appears as a low-signal-intensity structure on T1- and T2-weighted sequences and can be clearly delineated from surrounding musculotendinous and retinacular components. In most cases, accessory imaging planes are not required, and the tendon can be traced continuously from origin to insertion with minimal artifact [5].

#### 4.1.2. Preoperative Imaging Utility

Although preoperative imaging is not routinely indicated for Type I configurations, it may be useful in procedures involving EHL transfer, graft harvesting, or revision surgery where precise knowledge of the tendon course is essential. Additionally, imaging may help differentiate true EHL from adjacent structures such as EHB or anomalous fascicles in cases of trauma or surgical scarring [15].

In elective reconstructions, confirmation of a single-tendon morphology can streamline intraoperative dissection and minimize operative time, especially when EHL is selected as a donor for tendon transfer in drop foot correction or extensor mechanism reconstruction [13,14].

### 4.2. Type II

#### 4.2.1. Type IIa

Dynamic US examination is particularly useful for Type IIa variants, enabling real-time visualization of the independent gliding motion of the accessory tendon slip. MRI offers detailed static visualization, with particular focus on the proximal phalanx insertion site [5,6].

#### 4.2.2. Type IIb

High-resolution 3 Tesla (3T) MRI is recommended for identifying the bifid accessory tendon slips characteristic of Type IIb. These may resemble pathological entities such as ganglion cysts or partial tendon tears, particularly on lower-field scanners. Multiplanar imaging and clinical correlation are crucial to avoid misdiagnosis [5].

#### 4.2.3. Type IIc

Type IIc involves an accessory tendon inserting onto the dorsal base of the first metatarsal. Imaging protocols should include this region using wide-field-of-view MRI and multiplanar reconstruction. It is important to distinguish this slip from tendons such as the extensor digitorum longus or variant EHB [1,15].

#### 4.2.4. Unified Conclusion for Section 4.2.1, Section 4.2.2 and Section 4.2.3

Across all Type II variants, accurate imaging is essential due to the variable course and insertion of the accessory slip. One of the most common diagnostic challenges is misidentification of the accessory tendon as EHB, particularly when insertions are proximal or medial. Standardized protocols using high-resolution ultrasound and MRI enhance preoperative planning and reduce the risk of intraoperative errors.

### 4.3. Type III

#### 4.3.1. Advanced Imaging Techniques

Type III variants, characterized by three distinct tendinous slips with complex insertions, require the use of high-resolution imaging modalities for accurate diagnosis. High-field 3 Tesla (3T) MRI with multiplanar and 3D reconstruction capabilities provides the spatial resolution necessary to delineate the three separate tendon slips, their morphology, and relationships to adjacent structures. Similarly, 3D US offers dynamic and volumetric assessment, allowing visualization of tendon excursion and interaction with the EHB [6].

#### 4.3.2. Diagnostic Challenges

Due to the rarity and complexity of Type III, these tendinous configurations are often misinterpreted as traumatic injuries such as partial or complete tears, or as inflammatory masses like tenosynovitis or ganglion cysts on standard imaging. Misdiagnosis may lead to inappropriate surgical planning or unnecessary interventions. Therefore, it is critical to differentiate anatomical variants from pathological entities during preoperative evaluation, particularly when planning reconstructive surgery or tendon transfers involving the EHL complex [5,12].

Training and recognition (radiology).

Accurate recognition of EHL variants—particularly Type III—benefits from a structured imaging approach. We recommend:

1. Field of view & planes (MRI): ensure coverage from the extensor retinaculum to the first-metatarsal base and distal phalanx; include thin-slice axial and sagittal PD-fat-sat/T2-fat-sat. When available, add a thin-slice 3D isotropic sequence to reformat along the course of suspected accessory slips.
2. Dynamic high-frequency ultrasound: track the main EHL distally and actively follow each slip to its insertion; confirm independent glide of small accessory bands with resisted hallux dorsiflexion.
3. Landmark correlation: map suspected slips against EHB/EDL to avoid mislabeling an accessory EHL as EHB. Side-by-side assessment of their courses over the first ray reduces false positives.
4. Type III focus: small-caliber, obliquely oriented third slips are prone to partial-volume loss on MRI and to coalescing with EHB on US. Use short-axis “stop-frame” sweeps every few millimeters and verify continuity in two orthogonal planes.
5. Structured reporting: include (i) type label (I, IIa–c, III); (ii) insertion site(s) (distal phalanx/proximal phalanx/first-metatarsal base); (iii) relationship to EHB/EDL (crossing, blending, independence); (iv) dynamic behavior (independent glide/snapping); and (v) surgical note (e.g., “accessory slip likely encountered during dorsal approach; preserve/identify”). This targeted workflow reduces inter-observer variability, minimizes misidentification (especially vs. EHB), and improves preoperative planning for foot and ankle surgery.

For clinical contexts in which these imaging considerations apply, see Table 2.

## 5. Surgical Implications in Orthopedics

### 5.1. Type I

#### 5.1.1. Standard Surgical Techniques

Type I, the classical single-tendon configuration of the EHL, allows for standard dorsal foot approaches without modification. Its predictable anatomy facilitates uncomplicated surgical exposure and minimizes technical difficulty [1]. For a concise overview of major clinical conditions involving the EHL and their variant-specific implications, see Table 2.

#### 5.1.2. Tendon Reconstruction and Transfers

Thanks to its consistent morphology and stable distal insertion, Type I EHL is particularly well-suited for graft harvesting and tendon transfer procedures. Its unbranched course enables safe mobilization with minimal risk to adjacent structures, supporting effective reconstruction in cases such as foot drop or hallux deformity [13,14].

### 5.2. Type IIa

#### 5.2.1. Potential Surgical Pitfalls

In Type IIa variants, the accessory tendon slip inserts near the EHB and may complicate intraoperative identification. If not carefully recognized, it can be inadvertently damaged or excluded from repair, potentially compromising hallux extension strength [6].

#### 5.2.2. Recommended Management

Preoperative imaging with high-resolution US and MRI is strongly recommended to accurately delineate the accessory slip’s course and insertion. Precise mapping aids surgical planning, ensuring preservation of the auxiliary tendon during dissection and reconstruction. Intraoperative identification should be performed carefully, with awareness of the possible presence of this variant to avoid unintended damage [5,6].

### 5.3. Type IIb

#### 5.3.1. Surgical Complexity

The bifid insertion characteristic of Type IIb increases operative complexity due to the presence of two closely apposed tendinous slips inserting near each other on the proximal phalanx. There is a heightened risk of inadvertently splitting or damaging both the EHL and the EHB tendons during dissection or repair [5,6]. This configuration demands meticulous surgical technique to preserve tendon integrity.

#### 5.3.2. Surgical Recommendations

Surgeons are advised to perform the procedure under magnification, using surgical loupes or an operating microscope to enhance visualization of the bifid tendon slips. Careful identification and separation of the individual slips prior to repair or transfer is essential to avoid compromising either tendon and to ensure optimal functional restoration [12].

### 5.4. Type IIc

#### 5.4.1. Deep Variant Surgical Challenges

Type IIc is characterized by an accessory tendon slip inserting onto the dorsal base of the first metatarsal, making it a deeper and less accessible variant during standard dorsal foot approaches. This anatomical depth complicates surgical exposure, increasing the difficulty of safe dissection and repair [6].

#### 5.4.2. Risks of Misidentification

The deep position and anatomical proximity of the accessory slip in Type IIc increase the risk of confusion with nearby tendons, such as EHB or extensor digitorum longus. Such misidentification may lead to incomplete repair or unintended transection, compromising surgical outcomes [15].

#### 5.4.3. Surgical Recommendations

A wider surgical approach is recommended to adequately visualize and preserve this deep accessory slip. Preoperative three-dimensional imaging and meticulous surgical planning are essential to minimize the risk of iatrogenic injury. Utilization of advanced imaging modalities such as 3D MRI or ultrasound is strongly advised to map the exact location and trajectory of the accessory tendon prior to surgery [6].

### 5.5. Type III

#### 5.5.1. Highest Complexity Variant

Type III represents the most anatomically complex variant of the EHL tendon, featuring three distinct tendinous slips with separate insertions. This complexity necessitates particular caution during reconstructive surgeries, excision of ganglion cysts, and repair of traumatic tendon injuries [1].

#### 5.5.2. Operative Risks

Failure to recognize and preserve all three slips intraoperatively can result in incomplete reconstruction, persistent hallux dysfunction, or postoperative pain. The lateral slip’s fusion with the EHB further complicates dissection, increasing the risk of inadvertent injury to adjacent tendons or neurovascular structures [10,12].

#### 5.5.3. Surgical Recommendations

Surgical intervention for Type III variants should only be undertaken with comprehensive preoperative 3D imaging, such as high-resolution MRI or 3D US, to accurately map the tendinous anatomy. Reconstruction may require autografts to restore functional integrity of all slips, and meticulous surgical technique to minimize complications and optimize outcomes [1,6].

### 5.6. Surgical Access Challenges

#### 5.6.1. Type I

Surgical access challenges for Type I variants are minimal due to the straightforward and superficial course of the EHL tendon. Standard dorsal approaches to the foot are typically sufficient to expose the tendon in its entirety. Procedures can be performed with the patient in the usual supine position, and conventional longitudinal incisions over the dorsum of the first ray provide adequate visualization without necessitating extension or modification of the surgical field [1,13].

#### 5.6.2. Types IIa–IIc

Surgical exposure of Type II variants (IIa, IIb, IIc) requires more extensive incisions than Type I due to the presence of accessory tendinous slips with variable and sometimes deep insertions. The increased anatomical complexity necessitates thorough preoperative imaging assessment, typically involving high-resolution US and MRI, to precisely map the accessory tendons and their relation to critical neurovascular structures [6,15].

The elongated surgical approach aims to provide adequate visualization and mobilization of all tendinous slips while minimizing iatrogenic injury. Importantly, the risk of damaging dorsal digital nerves or their branches increases, especially in IIc variants where accessory slips insert near or on the first metatarsal base, a region densely innervated by sensory nerves. Careful dissection under magnification is advised to preserve nerve integrity and reduce postoperative complications such as neuropathic pain or sensory deficits [5].

#### 5.6.3. Type III

Due to the intricate anatomy of Type III variants, featuring three distinct tendinous slips with varied insertions, surgical access often requires more extensive exposure. This may involve a larger dorsal incision or even two independent surgical approaches to adequately visualize and manage each slip [1].

Intraoperative tendon tracking techniques are highly recommended to minimize the risk of inadvertently damaging accessory slips. Use of real-time ultrasound guidance or neurostimulation can aid surgeons in identifying tendon paths and preserving the integrity of all components [12]. Such advanced intraoperative modalities help reduce complications, improve surgical precision, and enhance functional outcomes in these complex cases.

### 5.7. Type-Specific Surgical Recommendations

The detailed surgical approach for specific types of the EHL tendon variants allows optimizing surgical outcomes and minimizing complications. Knowledge of anatomical variants along with their imaging and surgical implications is essential for orthopedic surgeons performing procedures on the dorsum of the foot—Table 1.

**Table 1:** Type-specific surgical recommendations for EHL tendon variants (Olewnik classification, Types I–III; IIa–IIc).

Type	Surgical Recommendations	References
I	Standard dorsal approach; reliable for autologous tendon graft harvesting.	(Carda et al. [13]; Olewnik et al. [1])
IIa	Preoperative identification of the accessory slip adjacent to EHB; during exposure, identify and preserve the slip to avoid inadvertent transection.	(Boyd et al. [5]; Olewnik et al. [1])
IIb	Use magnification (micro-surgical loupes). Handle bifid slips as separate structures with individual suturing/tenodesis as indicated.	(Boyd et al. [5])
IIc	Extend the dorsal exposure toward the base of the first metatarsal; prepare for reinsertion/tenodesis at 1MT base and protect EHB during dissection.	(Aparisi Gómez et al. [15]; Olewnik et al. [6])
III	Plan with high-resolution MRI/ultrasound (±3D isotropic MRI); expect multiple fine slips, perform meticulous dissection; consider autograft reconstruction and, when complex, involve a reconstructive team.	(Olewnik et al. [6])

Footnote. Types follow the Olewnik classification (I–III; IIa–IIc). Abbreviations: EHB—extensor hallucis brevis; 1MT—first metatarsal; US—ultrasound; MRI—magnetic resonance imaging.

### 5.8. EHL in Major Diseases and Surgical Scenarios

Beyond type-specific anatomy, several common conditions involve the EHL directly or place it at risk during exposure and reconstruction. These include traumatic lacerations and attritional or iatrogenic ruptures after first-ray procedures [6], hallux extensus or clawing with dorsal pain or snapping over the first ray [5,16], postoperative adhesions and tethering limiting tendon glide [6], and scenarios requiring tendon balancing, Z-lengthening, or tendon transfer or augmentation [13]. Recognizing whether the tendon is single, bifid, or accompanied by accessory slips (Types I–III; IIa–IIc) refines preoperative planning, prevents inadvertent transection, and guides variant-aware repair, tenodesis, or transfer strategies [1,6].

Variant anatomy also influences imaging–exam concordance and intraoperative decision-making. Accessory or bifid slips may mask partial functional loss, mimic the extensor hallucis brevis on imaging, or create friction and snapping across the dorsum of the first ray [5,6]. A structured approach, combining dynamic high-frequency ultrasound to track each slip to its insertion with thin-slice MRI including first-ray coverage and course mapping, helps define the operative plan and anticipate the need for separate handling of slips, protection of EHB and EDL, and, in complex Type III patterns, potential graft planning [13,15,16]. A concise summary of major EHL-related conditions with variant interplay, imaging pointers, and surgical notes is provided in Table 2.

**Table 2:** Major clinical conditions involving the extensor hallucis longus (EHL): variant interactions, imaging, and surgical notes.

Condition	Typical EHL Involvement	Variant Interplay (Type)	Imaging Pointers (US/MRI)	Surgical Notes
Traumatic laceration/rupture	Direct discontinuity; loss of active hallux dorsiflexion.	Accessory/bifid slips may mask partial function (IIa–IIc, III).	Dynamic US to track each slip to insertion; thin-slice axial/sagittal MRI.	Identify and repair all slips; protect EHB; consider graft if chronic.
Iatrogenic injury after first-ray surgery (e.g., hallux valgus)	Inadvertent transection or adhesions during dorsal exposure.	Extra slips increase risk during exposure (IIa–IIc).	Discordance between exam and “single-slip” imaging suggests a variant.	Variant-aware dissection; Z-lengthening or tenodesis if imbalance.
Hallux extensus/claw hallux	Overpull or imbalance; dorsal pain/snapping.	Accessory slip to PP or 1MT base can accentuate deformity (IIa/IIc).	Dynamic US for snapping band; include 1MT base in MRI FOV.	Balancing/lengthening; consider tenodesis of accessory slip.
Snapping tendon over first ray	Friction of accessory band across dorsum.	Most often IIa/IIc; occasionally III.	Dynamic US to reproduce snap; MRI for course mapping.	Release or reroute accessory slip; preserve main EHL.
Chronic adhesions/tethering	Limited glide after trauma/surgery.	Any type; complexity rises with multiple slips.	US for reduced excursion; MRI for scarring planes.	Adhesiolysis; ensure independent glide of each slip.
Anterior tibialis tendon (TAT) rupture (salvage)	EHL considered for transfer/augmentation.	Prefer single robust slip (I); complex in II–III.	Confirm caliber and independence of slips on US/MRI.	Plan harvest preserving hallux function; consider EDL/EHB balance.
Hallux varus/valgus procedures (balancing)	Z-lengthening/tenodesis for balance.	Extra slips may require separate handling (IIb/IIc, III).	Map slips vs. EHB/EDL preoperatively.	Protect EHB; suture slips independently; avoid over-lengthening.
Variant-related impingement at first-metatarsal base	Accessory to 1MT (IIc) causes focal pain.	IIc.	Include 1MT base on MRI; dynamic US for focal tenderness.	Local decompression or tenodesis; preserve main slip.

Footnote. Abbreviations: PP—proximal phalanx; 1MT—first metatarsal; EHB—extensor hallucis brevis; EDL—extensor digitorum longus; US—ultrasound; MRI—magnetic resonance imaging.

## 6. Comparative Overview of Existing Classifications

Although numerous anatomical studies have described the variability of the EHL tendon, very few have proposed structured classification systems that are clinically useful. Most early reports were descriptive, focusing either on distal insertion patterns or isolated anomalies, with limited reproducibility or surgical application. In this context, the classification proposed by Olewnik et al. [1] represents a significant advancement, offering a reproducible, imaging-compatible, and surgically oriented system. This section provides a comparative analysis of existing literature.

### 6.1. Early Insertion—Based Typologies

One of the earliest structured attempts to categorize EHL morphology was by Al-Saggaf [2], who examined 50 lower limbs and described four types of tendon insertions:

- Type I—insertion onto the base of the distal phalanx,
- Type II—additional fibers inserting into the capsule of the interphalangeal joint,
- Type III—additional insertion on the base of the proximal phalanx,
- Type IV—insertion into the extensor expansion.

While these types were numerically labeled, the system remained limited to the description of distal tendon endpoints. It did not address tendon trajectory, the number of slips, or relationships with adjacent structures. Moreover, the lack of imaging or surgical correlation makes this system poorly applicable in clinical practice.

### 6.2. Observational Anatomical Reports

Park et al. [4], in a cadaveric study of 158 Korean feet, identified three morphological types of the EHL tendon based on the presence and number of accessory tendinous slips. In addition to the main classification (Types I–III), the authors proposed a functional subclassification (Types 1K–3K) for accessory EHL tendons (ATEHL), depending on their direction and relationship to the EHB. These variants were observed in nearly one quarter of specimens, with Type II (one accessory tendon) and Type III (two accessory tendons) being less frequent but clinically notable. Although the study enhanced morphological understanding and suggested potential biomechanical implications such as EHL-EHB imbalance or hallux valgus predisposition, it did not establish a reproducible classification for surgical application.

Li et al. [17] examined 50 lower limbs and reported a 100% prevalence of an accessory EHL tendon. They proposed a morphological typology based on the directional course of this accessory tendon: Type I (parallel), Type II (oblique medial), and Type III (oblique lateral). While their study highlighted the possible stabilizing function of the ATEHL in resisting hallux valgus progression, it lacked a framework for operative planning and did not correlate findings with imaging modalities.

### 6.3. The Olewnik Classification: A Functional Framework

The classification introduced by Olewnik et al. [1,6] represents the first large-scale anatomical and imaging-based system dedicated to the EHL tendon. It defines three main types based on the number and course of tendinous slips, further subdividing Type II into subtypes IIa, IIb, and IIc according to accessory slip insertion. This system was derived from the analysis of 102 lower limbs and supplemented by high-resolution MRI and ultrasound validation.

In contrast to earlier descriptions, the Olewnik classification has multiple advantages:

- Comprehensiveness—It incorporates both the number of tendinous slips and their anatomical relationships with other dorsal foot structures, such as EHB and the first metatarsal.
- Scalability—It allows for practical subdivision into subtypes, enabling precise identification of less common variants.
- Radiological Relevance—The classification is grounded in US findings, ensuring compatibility with modern diagnostic workflows.
- Surgical Utility—Each type is associated with specific surgical risks and implications, including tendon harvest, deformity correction, and operative exposure.
- Reproducibility—With a cadaveric basis and imaging confirmation, the classification provides reliable application across anatomical and clinical contexts.

These attributes make it the first system to combine academic rigor with practical orthopedic relevance.

### 6.4. Comparative Overview of EHL Tendon Classifications—Table 3

The main classification attempts are summarized below, outlining their methodological basis, scope, and limitations.

**Table 3:** Comparative Overview of EHL Tendon Classifications.

Author(s)	Year	Study Type	Classification Proposed	Limitations
Al-Saggaf [2]	2003	Cadaveric	Partial (insertion-based only)	Lacks imaging or surgical applicability
Park et al. [4]	2022	Cadaveric	No	Functional insight only, no reproducible classification
Li et al. [17]	2021	Cadaveric	Directional subtypes only	No imaging, no surgical relevance
Olewnik et al. [1,6]	2019–2020	Cadaveric + Imaging	Yes—Types I–III (with IIa–c)	Comprehensive, validated, clinically applicable

### 6.5. Comparative Clinical Relevance of EHL Classifications

Although earlier studies such as those by Park et al. [4] and Li et al. [17] offered valuable anatomical insights into the EHL tendon, their proposed systems lack the consistency, imaging correlation, and surgical relevance required for standardized clinical use. Park’s cadaveric classification, while proposing both morphological and functional subtypes of accessory EHL tendons, remains largely observational and lacks integration with preoperative imaging or operative protocols. Similarly, Li’s direction-based categorization of accessory slips highlights their potential functional role but fails to translate into a reproducible or radiologically supported framework.

In contrast, the classification introduced by Olewnik et al. [1,6] stands out for its comprehensive design and clinical utility. It systematically stratifies EHL tendon morphology based on the number and insertion of tendinous slips, correlates anatomical patterns with high-resolution US and MRI findings, and explicitly links each variant to specific surgical approaches and potential complications.

This integrative framework offers several key advantages:

- Imaging Compatibility: Each type and subtype is validated by ultrasonography and MRI, ensuring real-world diagnostic feasibility.
- Surgical Applicability: The classification is directly translatable to operative decision-making, including tendon harvest, deformity correction, and intraoperative navigation.
- Reproducibility: Derived from a large cadaveric sample and confirmed radiologically, the system enables reliable use across anatomical, radiological, and surgical settings.

The Olewnik classification is currently the only system to unify anatomical, imaging, and procedural dimensions of EHL variability, making it the most robust and clinically actionable framework available. Given its cadaveric foundation, radiologic correlation, and direct surgical applicability, the Olewnik classification stands as the most mature framework currently available for guiding operative decision-making in hallux-related pathologies.

## 7. Future Directions

Despite recent advances in the anatomical and surgical understanding of EHL tendon morphology, several areas remain open for further investigation and refinement of clinical protocols. Based on the current classification and comparative findings, the following research directions are proposed:

### 7.1. Prospective Clinical Validation

To establish the full clinical utility of the Olewnik classification, prospective studies are needed to correlate each morphological type with intraoperative findings, postoperative outcomes, and complication rates. Such studies could clarify whether specific EHL variants predispose to surgical challenges or affect graft harvest efficiency [1,6].

### 7.2. Radiological Standardization and AI-Assisted Identification

Although MRI and US have been shown to detect EHL variants with high resolution [4,6], there is a need for standardized imaging protocols particularly for preoperative planning in tendon transfer and deformity correction. Future research may also explore the role of artificial intelligence and machine learning in automatic identification of EHL subtypes using annotated datasets, especially in high-volume centers where radiological workflows may benefit from semi-automated annotation of tendon variants [17].

### 7.3. Biomechanical and Functional Assessment

The biomechanical implications of EHL tendon variants have yet to be quantitatively assessed. Experimental models and cadaveric simulations may help determine whether accessory slips alter toe extension strength, joint torque, or forefoot pressure distribution especially in complex patterns such as Types IIb and III [2,4]. These insights could refine surgical indications and postoperative rehabilitation strategies.

### 7.4. Pediatric and Developmental Studies

EHL tendon morphology during growth remains underexplored. Longitudinal studies in pediatric populations or fetal anatomical series may offer insight into the ontogeny of accessory tendinous slips and explain the developmental basis for the variant patterns described in adults [6,17].

### 7.5. Integration into Surgical Education and Classification Systems

Incorporating the EHL classification into surgical training curricula, operative atlases, and anatomical databases such as Terminologia Anatomica or the FMA could enhance awareness and promote widespread adoption. Furthermore, consensus-driven refinements through multicenter studies may yield an internationally recognized system with predictive surgical value [1,4].

## 8. Summary and Conclusions

The classification of extensor hallucis longus (EHL) tendon variants proposed by Olewnik et al. offers orthopedic surgeons a valuable framework to anticipate intraoperative challenges and reduce complications associated with unrecognized anatomical variability. By delineating distinct morphological types, this system facilitates tailored surgical planning and enhances the precision of reconstructive and tendon transfer procedures.

Imaging modalities, particularly high-resolution ultrasound and magnetic resonance imaging, play an indispensable role in the preoperative identification of EHL variants. Routine incorporation of variant-specific imaging assessment is recommended before surgical interventions involving the dorsal foot to ensure accurate diagnosis and appropriate operative strategy.

To fully realize the clinical utility of this classification, further studies correlating EHL variant types with surgical outcomes are necessary. Additionally, development of decision-making algorithms that integrate anatomical subtype, imaging findings, and patient-specific factors would aid in optimizing treatment strategies. Dissemination and adoption of this classification among foot and ankle surgeons are critical steps toward improving patient care in this anatomically complex region.

## Data Availability

No new data were created or analyzed in this study.
